# Interleukin 15 in Cell-Based Cancer Immunotherapy

**DOI:** 10.3390/ijms23137311

**Published:** 2022-06-30

**Authors:** Yang Zhou, Tiffany Husman, Xinjian Cen, Tasha Tsao, James Brown, Aarushi Bajpai, Miao Li, Kuangyi Zhou, Lili Yang

**Affiliations:** 1 Department of Microbiology, Immunology & Molecular Genetics, University of California, Los Angeles, CA 90095, USA; zzydcat@ucla.edu (Y.Z.); tiffanyhusman@g.ucla.edu (T.H.); xicen@ucla.edu (X.C.); tytsao@ucla.edu (T.T.); brownjimw0@gmail.com (J.B.); abajpai2023@ucla.edu (A.B.); ericmli0507@ucla.edu (M.L.); kuangyizhou@g.ucla.edu (K.Z.); 2 Eli and Edythe Broad Center of Regenerative Medicine and Stem Cell Research, University of California, Los Angeles, CA 90095, USA; 3 Jonsson Comprehensive Cancer Center, David Geffen School of Medicine, University of California, Los Angeles, CA 90095, USA

**Keywords:** interleukin 15, cancer, immunotherapy, T cell, NK cell, chimeric antigen receptor, engineering, adoptive cell transfer

## Abstract

Cell-based cancer immunotherapy, such as chimeric antigen receptor (CAR) engineered T and natural killer (NK) cell therapies, has become a revolutionary new pillar in cancer treatment. Interleukin 15 (IL-15), a potent immunostimulatory cytokine that potentiates T and NK cell immune responses, has demonstrated the reliability and potency to potentially improve the therapeutic efficacy of current cell therapy. Structurally similar to interleukin 2 (IL-2), IL-15 supports the persistence of CD8^+^ memory T cells while inhibiting IL-2-induced T cell death that better maintains long-term anti-tumor immunity. In this review, we describe the biology of IL-15, studies on administrating IL-15 and/or its derivatives as immunotherapeutic agents, and IL-15-armored immune cells in adoptive cell therapy. We also discuss the advantages and challenges of incorporating IL-15 in cell-based immunotherapy and provide directions for future investigation.

## 1. Introduction

Immunotherapy has advanced the field of cancer research and oncology with revolutionary technologies and numerous clinical trials. Adoptive cell therapies, wherein immune cells isolated from patients or healthy donors are infused into patients after ex vivo expansion and engineering, have demonstrated promising results in treating certain subsets of B cell leukemia or lymphoma [1]. This is particularly true when used in combination with modified T cell receptors (TCRs) or chimeric antigen receptors (CARs), which facilitate anti-tumor efficacy and yield remarkable clinical responses [1,2,3]. Currently, there are six CAR T therapies approved in the United States (US) for treating B cell lymphoma, follicular lymphoma, mantle cell lymphoma, multiple myeloma (MM), and acute lymphoblastic leukemia (ALL). However, cell-based immunotherapy, such as CAR-T therapy, struggles with poor proliferation and persistence in treating certain malignancies, especially in treating solid tumors. Specifically, the inhibitory tumor microenvironments (TME) can suppress TCR and costimulatory signals, restrict cell trafficking, and inactivate CAR-T cells. Many strategies have been investigated to improve CAR-T function, such as utilizing stimulatory cytokines to increase the survival and expansion of immune cells and reverse the immunosuppressive tumor microenvironment [4].

Interleukins (ILs), a group of cytokines primarily expressed and secreted by leukocytes, play a significant role in promoting the development, differentiation, and function of immune cells, such as T and NK cells. Most interleukin cytokines exert multifarious roles in the anti-tumor process. Many studies have investigated the co-expression of one or more cytokines (such as IL-2, IL-7, IL-12, IL-15, IL-18, IL-21, and IL-23), or the combination of their receptors to generate gene-edited interleukin-armored immune cells against cancer. Of note, Interleukin-2 (IL-2) is one of the most well-studied and became the first immunotherapy that was approved by the US Food and Drug Administration (FDA) for cancer treatment nearly 30 years ago [5]. Administration of IL-2 in patients with metastatic melanoma and renal carcinoma demonstrated regression of metastasis and durable complete responses [6,7]. Strategies incorporating IL-2 into ex vivo expansion protocols have been investigated for immune cells, such as tumor-infiltrating T (TILs), CAR-T, and NK cells, to promote their activation and proliferation. However, IL-2-expanded immune cells are more differentiated, which can shorten their long-term persistence and survival. Consequently, researchers have begun exploring other cytokines, such as IL-7, IL-15, and IL-21, to overcome the drawbacks and improve the quality of cellular products.

Interleukin 15 (IL-15) was discovered in 1994 due to its similar functionality to IL-2 in regulating T-cell and natural killer (NK) cell proliferation as well as inducing B-cell immunoglobulin synthesis [8]. IL-2 and IL-15 share the β chain and γ chain in their receptors leading to similar downstream signaling effects [1,5]. Although structurally similar to IL-2, IL-15 displays distinct characteristics in vivo. IL-2 is required for the development and maintenance of regulatory T-cells (Treg), while IL-15 has no effect on Tregs and is a key supporter of NK cell and memory CD8^+^ T-cell survival [5,9,10,11,12]. The specific receptor components IL-2Rα and IL-15Rα may contribute to their differing functions [5]. Because of these benefits, IL-15 was ranked first for having the greatest potential in cancer immunotherapy by the US National Cancer Institute in 2008 [13]. However, a short half-life (less than 1 h in humans) and tight posttranscriptional regulation limit the application of IL-15 [14]. Extensive efforts have been expended to overcome these challenges. The most common approach is by increasing the molecular size to reduce the renal elimination or by targeting the neonatal Fc receptor, exemplified by hetIL-15 (NIZ985), hetIL-15Fc, receptor-linker-IL-15 (RLI), N-803 (ALT-803), and NKTR255 [15,16]. The efficacy and safety of IL-15 derivatives have been evaluated in clinical trials either as a monotherapy or in combination with chemotherapy or immune checkpoint blockades. Additionally, overexpression of soluble IL-15 (sIL15) or membrane-bound IL-15 (mbIL15; IL-15Rα/IL15 complex) to generate IL-15-armored immune cells has demonstrated enhanced anti-tumor effects and cell persistence [17,18]. In this review, we discuss the biology of IL-15 and its receptors, IL-15 and its derivative products for cancer immunotherapy, as well as the adoptive transfer of IL-15-armored immune cells in cell therapies. We also present the limitations and future directions of incorporating IL-15 in cell-based cancer immunotherapy.

## 2. Biology of IL-15 and Its Receptor

Interleukin-15 (IL-15) was originally discovered simultaneously in 1994 by two research groups for its ability to mimic IL-2-stimulated T cell proliferation [19,20]. The cytokine was initially found to be secreted by a T-cell leukemia cell line (HuT-102) as well as a kidney epithelial cell line (CV-1/EBNA) [21] and was termed a “T-cell growth factor”. IL-15^−/−^ and IL-15Rα^−/−^ mice suffered from lymphopenia. They showed a great deficiency in CD8^+^ T, natural killer (NK) and natural killer T (NKT) cells, as well as defects in lymphocyte homing, which could be reversed with the administration of IL-15, demonstrating that IL-15 promotes the proliferation and homeostasis of specific lymphoid lineages [8,12].

**Figure 1:**
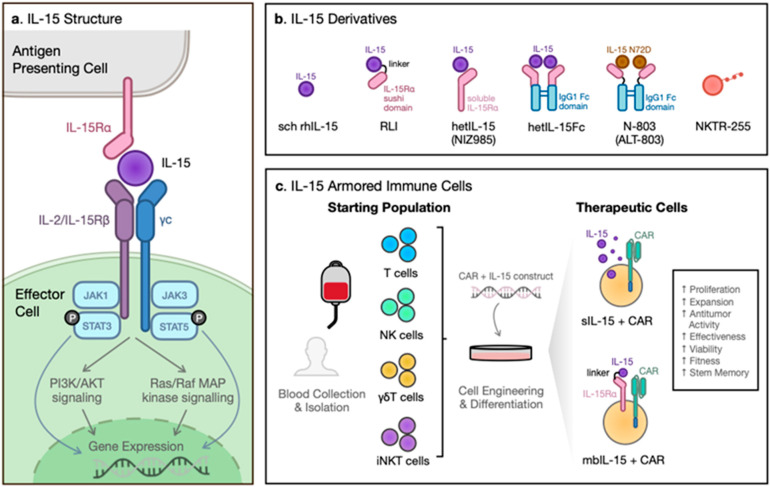
IL-15 and its therapeutic applications. (**a**) The structure and downstream signaling of IL-15 and its receptor complex. IL-15 is trans-presented on antigen-presenting cells by IL-15Rα, which interacts with the β chain (IL-2/15Rβ) and common γ chain (γc) complex on effector cells. Upon activation, the β and γc receptor triggers the intracellular signaling of the Janus kinase pathway, which stimulates the signal transducer and activator of transcription (STAT) proteins downstream. The phosphorylated STATs relocate to the nucleus, modifying gene expression. (**b**) IL-15 derivatives and their structure. From left, *Escherichia coli*-derived IL-15 monomer (sch rhIL-15), Receptor-Linker-IL-15 fusion protein consisting of IL-15 linked to the sushi domain of IL-15Rα (RLI), IL-15 heterodimer with soluble IL-15Rα (hetIL-15/NIZ985), another form of hetIL-15 where C-terminus of IL-15Rα is linked to the Fc region of human IgG1 (hetIL-15Fc), N72D mutant and human Il-15Rα sushi domain-Fc fusion protein (N-803/ALT-803) and polyethylene glycol-conjugate of rhIL-15 (NKTR-255). (**c**) IL-15 armored immune cells. The starting population of T, NK, γδT, iNKT cells collected from healthy donors are engineered and differentiated into therapeutic cells armored with soluble IL-15 (sIL-15) and CAR, or membrane-bound IL-15 (mbIL-15) and CAR. Co-expressing IL-15 or mbIL-15 with CAR enhances immune cells proliferation and expansion, increases anti-tumor activity and effectiveness, improves viability and cellular fitness, and increases stem memory.

IL-15, a 14–15 kDa glycoprotein encoded on the human chromosome 4q31 [22], is expressed in various cell types. IL-15 mRNA is widespread in a broad range of tissues, including hematopoietic cells (such as monocytes, macrophages, and dendritic cells) and non-hematopoietic cells (such as keratinocytes, fibroblasts, nerve cells, skeletal muscle, and epithelial cells) [8], while IL-15 protein is only detectable in a restricted population of cells. As part of the diverse four α-helix bundle family of cytokines, IL-15 shares the common gamma (γc) chain receptor with other members such as IL-2, IL-4, IL-7, IL-9, and IL-21 [23,24]. Within the family, IL-15 shares the most common features with IL-2, including the IL-2 receptor beta (IL-2Rβ or CD122) and common gamma receptor (γc or CD132) [25] (Figure 1a). Despite these similarities, IL-15 has its own unique high-affinity IL-15 receptor alpha (IL-15Rα) for ligand specificity (1000-fold higher compared to IL-2Rα) [22,26,27]. IL-15Rα is an atypical cytokine receptor with hydrophilic residues surrounding the four α-helix hydrophobic cores [26]. Its sushi domain mediates the remarkable high binding affinity of the strongly negatively charged IL-15 binding region to the positively charged surface of IL-15Rα through ionic interactions [22,26], which leads to a stable complex that is involved in the presentation of IL-15 [28]. Notably, IL-15Rα is not only present as a membrane-bound receptor but has also been discovered in a soluble form, generated by the cleavage of the transmembrane receptor, which can serve as an inhibitor in a protective mechanism against excessive IL-15 activity [29]. In addition, many naturally occurring isoforms of IL-15 and IL-15Rα have been uncovered since the protein’s discovery in 1994. Different isoforms contribute to various functions within the cell, ranging from regulating internal signal transduction to increasing soluble cytokine secretion (Table 1).

Although the majority of IL-15 signaling involves the IL-15/IL-15Rα complex, IL-15 is also able to bind to the IL-2/IL-15Rβ/γc alone but does so with a lower binding affinity [25]. Upon activation, the β and γc receptor triggers the intracellular signaling of the Janus kinase (JAK) pathway, which stimulates the signal transducer and activator of transcription (STAT) proteins downstream (Figure 1a). The phosphorylated STATs relocate to the nucleus, modifying gene expression [23]. Notably, unlike IL-2, which requires the IL-2α unit for IL-2Rβ/γc signaling, IL-15 can bind the IL-15β and γc receptor and stimulate downstream signaling in the absence of the IL-15α unit [22].

## 3. Functionality of IL-15

IL-15, recognized as a potent immunostimulatory cytokine, potentiates innate and adaptive immune responses. IL-15 proves to be a promising candidate for cancer immunotherapy due to its functional similarity to IL-2 with several added benefits, including the lack of stimulation on regulatory T cell populations, reduced activation-induced cell death (AICD), and lower toxicity [33] (Table 2). IL-15 does not stimulate the population of regulatory T cells (Tregs) due to the lack of interaction between IL-15 and IL2-Rα on the cell surface of Tregs [12]. Tregs are notorious for inhibiting anti-tumor responses by penetrating the tumor microenvironment and releasing inhibitory cytokines (TGF-β, IL-13, IL-35), disrupting immune checkpoint-related pathways, and preventing antigen-presenting cell (APC) maturation. IL-2 at low doses causes the Treg population to expand, which dampens the overall anti-tumor effect and enhances peripheral tolerance [5,30]. Unlike IL-2, IL-15 suppresses AICD, reducing apoptosis and increasing T-cell persistence [5,9,11]. Moreover, IL-15 therapy is less toxic. Patients treated with IL-2 at high doses developed grade 3 and 4 adverse events (AEs) such as neurotoxicity, heart failure, and severe capillary leak syndrome (CLS) [45,46]. In contrast, treatment with IL-15 at high doses only resulted in mild CLS that led to grade 1 and 2 AEs such as fever, chills, rigors, and transient neutropenia [1,47]. Altogether, the advantages IL-15 has over IL-2 indicate that it could be a highly effective and safe cytokine for cancer therapy.

On the other hand, IL-15 is recognized to play an indispensable role in NK cell development and homeostasis. Deficiency in IL-15 or any of its receptor subunits resulted in a paucity of mature NK cells in mice. In humans, IL-15 is not directly involved in hematopoietic stem cell (HSC) commitment to NK progenitor cells, but it plays a critical role in regulating the development of mature CD56^+^ NK cells [48,49]. In a humanized mouse model, the development of human NK cells was shown to follow a process of CD56^+^CD16^−^KIR^−^ to CD56^−^CD16^+^KIR^−^, and then to CD56^−^CD16^+^KIR^+^, which required the presence of human IL-15 [50]. IL-15 is also a pro-inflammatory cytokine, influencing the function and survival of other immune cells in addition to T and NK cells [8,51]. IL-15 stimulates activated B lymphocytes and enhances their secretion of immunoglobulins [8,52]. For neutrophils, IL-15 not only serves as a suppressor of neutrophil apoptosis via the anti-apoptotic Mcl-1 protein and several kinase signaling pathways, but also activates phagocytosis and anti-microbial responses [53,54]. IL-15 is an apoptosis suppressor and a growth factor for mast cells [55]. In monocytes, IL-15 has the effect of increasing phagocytosis and the secretion of cytokines such as IL-6, IL-8, and TNFα [8]. Furthermore, IL-15 induces the maturation of dendritic cells and increases their expression of CD86, CD40, and MHC I as well as IFN-γ secretion [8,56]. Besides acting on immune cells, IL-15 also protects epithelial cells, keratinocytes, hepatocytes, and fibroblasts from apoptosis, induces angiogenesis with endothelial cells, and supports the survival of neurons [8].

## 4. Administration of IL-15 and Its Derivatives for Cancer Immunotherapy

Since its discovery in 1944, researchers have investigated methods to utilize IL-15’s immunomodulatory abilities to promote anti-tumor responses against cancers. Recombinant human IL-15 (rhIL-15) is an *Escherichia coli*-derived IL-15 monomer that has demonstrated pre-clinical success in several models and holds several advantages over IL-2 [57]. In lung adenocarcinoma (LA795) transplantable tumor mice models, tumor-bearing mice treated with rhIL-15 demonstrated a significantly reduced tumor load and prolonged survival compared to rhIL-2, suggesting that rhIL-15 had a superior anti-tumor effect at an equivalent dosage level [58]. The rhIL-15-induced tumor regression and decreased metastasis were also demonstrated in several other murine cancer models, including B16 melanoma, M38 colon carcinoma, TC-1 carcinoma, and lymphomas. The safety of rhIL-15 administration was proved in Rhesus macaques, where three different dosing methods (intravenous infusion, continuous intravenous infusion, and subcutaneous injection) were all tested [59]. rhIL-15 administration in another Rhesus macaque model significantly expanded memory CD8^+^ T cells and NK cells [60]. These promising results led to the first-in-human clinical trial of rhIL-15 in metastatic melanoma and renal carcinoma patients in 2015 [61] (ClinicalTrials.gov Identifier: NCT01021059). The safety and maximum tolerated dose were determined by administration via intravenous bolus (IVB) infusion over 12 consecutive days. rhIL-15 administered by bolus infusions in patients with metastatic melanoma or renal cancer revealed an efflux of NK and memory CD8^+^ T cells as well as altered homeostasis of NK cells and γδ cells [61], with moderate levels of toxicity such as fevers and chills. Subcutaneous (s.c.) administration of rhIL-15 was better tolerated than IVB while still producing a significant increase in circulating NK and CD8^+^ T cells [62] (ClinicalTrials.gov identifier: NCT01727076).

Despite these results, there are still obstacles to overcome with rhIL-15 protein monotherapy. Immune cell expansion requires long-term and sustained IL-15 exposure at moderate concentrations [63,64]; however, sustaining soluble IL-15 (sIL-15) is difficult due to its short serum half-life and regulatory mechanisms [65]. The biostability of IL-15 is largely limited by the availability of IL-15Rα [16,33]. Therefore, many strategies have been utilized to overcome these barriers by synthesizing different IL-15 derivatives (Figure 1b).

### 4.1. hetIL-15 (NIZ985)

Most IL-15 detected in mice and human serums exist as a heterodimer with IL-15Rα [16]. Thus, co-expression of IL-15 and IL-15Rα allows for efficient bioactive secretion in vivo compared to rlIL-15 alone, due to the increased stability of the heterodimer [16]. In 2013, a hetIL-15 preparation was developed, comprised of IL-15 and IL-15Rα [32]. Compared to rhIL-15, administration of hetIL-15 maintained sustainable levels of plasma IL-15 for a robust expansion of NK and T-cells in mice [16,32]. In pre-clinical murine models of MC38 colon carcinoma and TC-1 epithelial carcinoma, hetIL-15 treatment resulted in delayed primary tumor growth, expansion of NK and CD8^+^ T cell tumoral infiltration, and an increased CD8^+^/Treg ratio. In addition, intra-tumoral NK and CD8^+^ T cells showed increased interferon-γ (IFN-γ) production, proliferation, and enhanced cytotoxic potential [66]. hetIL-15 treatment in Rhesus macaques delivered subcutaneously (s.c.) resulted in the persistence of plasma IL-15 with a half-life of ~12 h [67]. Human hetIL-15 is now licensed by Novartis and currently has ongoing clinical trials under the name NIZ985. As a monotherapy, patients with metastatic or unresectable solid tumors were given s.c. injections three times a week with dose escalation throughout the trial. Results showed that treatments were generally well-tolerated and significantly induced the production of circulating IL-5, TNF-β, and IFN-γ, as well as proliferating cytotoxic lymphocytes. However, NIZ985 still causes certain severe adverse events when applied at high doses necessary for reaching a therapeutic effect [47] (ClinicalTrials.gov Identifier: NCT02452268, Table 3).

### 4.2. hetIL-15Fc

hetIL-15Fc is a fully glycosylated form of hetIL-15 where the C-terminus of IL-15Rα is covalently linked to the Fc region of human IgG1 [16,33,34]. hetIL-15Fc showed superiority over rhIL-15 in several murine models, including B16 melanoma and chronic lymphocytic leukemia [35,68,69]. It was found that adding the IgG1 Fc domain to IL-15Rα significantly enhances the survival and proliferation of NK and memory-like CD8^+^ T cells compared to IL-15Rα alone [69]. Additionally, high levels of granzyme B expression were observed in the supernatants of hetIL-15Fc-treated NK cells expanded ex vivo [35]. In vitro and in vivo, hetIL-15Fc enhanced the activity and the proliferation of NK and CD8^+^ T-cells [69]. hetIL-15Fc is also shown to double the period required for an initial 50% decline of IL-15 serum levels and increases half-life from 50 to >120 min [69]. These characteristics of heIL-15Fc may be due to the mimicking of the endogenous trans-presentation of IL-15 by IL-15Rα on artificial antigen-presenting cells (αAPCs) to NK and CD8^+^/CD44^high^ T cells in immune response [69]. However, several studies have shown that some side effects, such as target cell death and cytokine-induced tissue damage, can occur due to the interaction between Fc and complement proteins or FcγRs [36].

### 4.3. N-803 (ALT-803)

Another IL-15 preparation termed N-803, formerly known as ALT-803, is an IL-15 super-agonist comprised of an IL-15 variant with a higher binding affinity (IL-15N72D) complexed with a human IL-15Rα sushi domain-Fc fusion protein [37,38,39]. Treatment of mice with N-803 showed a 25 h half-life and a 5–20-fold increase of in vivo bioactivity [37]. Additionally, a single dose of N-803 delivered intravenously (i.v.) eliminated established tumors and prolonged survival of multiple myeloma mice models [39,70]. In mice bearing subcutaneous B16F10 melanoma and CT26 colon carcinoma, tissue biodistribution analysis demonstrated significantly greater retention of N-803 in lymphoid organs compared to IL-15 [39,71]. Similarly, N-803 was shown to enhance NK cell cytotoxicity in vitro and in vivo against ovarian cancer and rescue functionality in NK cells derived from patient ovarian cancer ascites [71]. In cynomolgus monkeys, N-803 induced dose-dependent increases of NK, CD4^+^ and CD8^+^ memory T cells and pharmacokinetic analysis revealed the half-life as approximately ~7.2–8 h [39]. In the first phase, I clinical trial of N-803, patients with advanced melanoma, renal cell, non-small cell lung, and head and neck cancer were treated with SC or intravenous (IV) N-803 weekly for 4 consecutive weeks, every 6 weeks [72]. Results revealed a significant increase in NK cell expansion and cytotoxicity with lesser CD8^+^ T cell expansion, with treatments being well-tolerated with mostly grade 1 and 2 AEs [72]. In another study, hematologic malignancy patients who relapsed after allogeneic hematopoietic cell transplantation (allo-HCT) were treated with N-803. An increase in activation, proliferation, and expansion was observed in NK cells and CD8^+^ T cells without an increase in Tregs [73]. N-803 was generally well-tolerated without dose-limiting toxicities or treatment-emergent graft-versus-host disease [73].

### 4.4. RLI

Receptor-Linker-IL-15 (RLI), an IL-15 agonist fusion protein, comprises the IL-15Rα sushi domain fused to IL-15 through a 20 amino acid linker [16,31]. RLI binds to IL-15β/γc with an almost 20-fold higher affinity and is rapidly internalized after binding, resulting in an overall greater potency than IL-15 [31]. In B16F10 melanoma mouse models, RLI displayed a longer in vivo half-life and higher efficiency than IL-15 or IL-2 in reducing lung and liver metastasis and prolonging survival [74]. RLI was also efficient at reducing tumor growth and metastasis of human colon carcinoma in orthotopic nude mice models [74]. RLI demonstrated antimetastatic characteristics in 4T1 mammary carcinoma murine models and restored the balance between NK cells and neutrophils in the lung microenvironment [30]. Pharmacodynamic analysis revealed superior proliferative and cytotoxic functions on NK cells with RLI treatment compared to IL-15 alone [30]. RLI was then utilized to treat patients with Stage III/IV non-small cell lung cancer (NSCLC). These patients typically have decreased NK cell function, including down-regulation of the activating receptor, NKp30, which may be a mechanism by which tumors avoid NK cell cytotoxicity. Stimulating PBMCs from NSCLC patients with RLI showed that it systematically increased the expression of activating receptors NKp30, NKp44, CD107a, and intracellular TNF-α, indicating that RLI could rescue the functionality of NK cells in patients with NSCLC [30]. RLI is currently being investigated in a phase I/Ib clinical trial in patients with advanced and metastatic solid cancers.

### 4.5. NKTR-255

NKTR-255 is a polyethylene glycol-conjugate of rhIL-15 designed to have a high binding affinity to IL-15R and increased persistence [40]. Pre-clinical trials of NKTR-255 have demonstrated enhanced anti-tumor activity and survival as a monotherapy, as well as in combination with monoclonal antibodies (mAbs) and with CD19-CAR T cell therapies [40,75]. NKTR-255 administration in mice resulted in a 2.5-fold expansion of CD8^+^ T cells and a 2-fold expansion of NK cells [76], which was independent of trans-presentation. In humans, NKTR-255 was shown to increase granzyme-B expression in peripheral blood compared to precomplexed cytokines such as hetIL-15 [40]. These successful results led to a dose expansion and escalation study, a Phase I clinical trial of NKTR-255 alone and in combination with daratumumab or rituximab in patients with multiple myeloma (MM) or Non-Hodgkin’s lymphoma (NHC) [75] (ClinicalTrials.gov Identifier: NCT04136756). Since then, NKTR-255 has been included in multiple other clinical trials alone or in combination with other therapies (Table 3).

## 5. Adoptive Transfer of IL-15-Armored Immune Cells for Cancer Immunotherapy

Besides using IL-15 and its derivatives alone in cancer immunotherapy, IL-15 has also been incorporated into many adoptive cell therapies against cancer, specifically in combination with chimeric antigen receptor (CAR) engineering. In many recent studies, researchers have attempted to incorporate IL-15 not only in ex vivo precultures but also by integrating IL-15 and its receptor in CAR engineering (Figure 1c).

### 5.1. IL-15-Armored CAR T Cells

**Figure 2:**
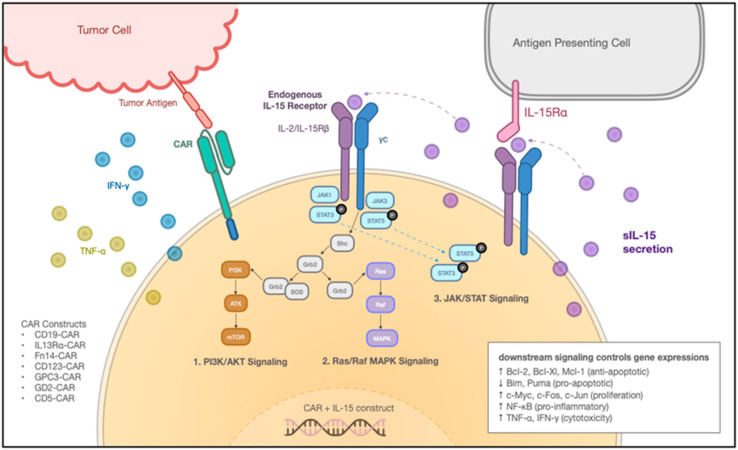
Mechanism of IL-15 Armored CAR T Cells. IL-15-armored CAR T cells mechanism of action. The engineered cells with the CAR and IL-15 transgene secrete soluble IL-15 (sIL-15), which either gets recognized directly by the endogenous IL-15 receptor or captured by neighboring Antigen Presenting Cells (APCs). After receiving the IL-15 signal, downstream signaling pathways include the PI3K/AKT signaling pathway, Ras/Raf MAPK signaling pathway, and JAK/STAT signaling pathway. These three pathways together control various gene expressions: upregulating Bcl-2, Bcl-Xl, Mcl-1 (anti-apoptotic), downregulating Bim, Puma (pro-apoptotic), upregulating c-Myc, c-Fos, c-Jun (proliferation), NF-κB (pro-inflammatory) and inducing the release of TNF-α, IFN-γ (cytotoxicity). On the left is a diagram representation of tumor recognition through CAR. CAR constructs include CD19-CAR, IL13Rα-CAR, Fn14-CAR, CD123-CAR, GPC3-CAR, GD2-CAR, and CD5-CAR et al.

CAR-engineering, which redirects T cells to specifically target the desired tumor-associated antigens (TAAs), has achieved successful outcomes in treating hematological malignancies. The successful complete responses in some patients were accompanied by the long-term surveillance of CAR T cells [77,78,79]. Considering the unique ability of IL-15 to maintain the homeostasis and proliferation of memory CD8^+^ T cells (Figure 2), various research groups explored the addition of IL-15 to CAR-T engineering. In Hoyos et al., researchers engineered T (iC9/CAR.19/IL-15 T) cells with a retroviral vector encoding anti-CD19 CAR, IL-15, and inducible caspase-9-based suicide gene (iC9) [80]. The IL-15-enhanced CAR T cells exhibited 10-fold expansion in vitro and 3- to 15-fold expansion in vivo, with reduced cell death and low PD-1 expression upon antigen stimulation. In the SCID lymphoma human xenograft model, the iC9/CAR.19/IL-15 T cells demonstrated greater effectiveness, along with better persistence and anti-tumor effects [80]. Similarly, Hurton et al. also tested a novel CAR construct, but with membrane-bound IL-15 (mbIL-15) fusion protein, consisting of IL-15 connected to IL-15Rα through a linker, to mimic the unique physiologic mechanism of IL-15 trans-presentation [17]. The mbIL15.CAR19 T cells preserved T memory stem cell phenotype and sustained T cell persistence in a CAR-independent manner. Compared to CAR19 T cells, the mbIL15.CAR19 T cells displayed similar gene profiling, cell expansion, and CAR expression under repetitive αAPC stimulation, but with the antigen withdrawn, the long-term persisting mbIL15.CAR19 T cells retained functional mbIL15 and effector responses [17]. The mbIL15 engineered CAR19 T cells showed superior anti-tumor activity with a strong memory stem cell phenotype in a humanized NSG mouse model.

Furthermore, IL-15 has also been integrated with other CARs for targeting different types of tumors, such as IL13Ra-CAR [81] and Fn14-CAR [82] for glioblastoma, GD2-CAR [83] for neuroblastoma, as well as Glypican-3 (GP3) CAR [84] for hepatocellular carcinoma (Table 4). Results consistently show that IL15-armored CAR T cells exhibit potent anti-tumor efficacy and enhanced persistence in vivo. Clinically, the transgenic expression of membrane IL-15 was first evaluated in a patient with B-cell acute lymphoblastic leukemia (B-ALL) after the failure of the first two CD19-CAR T and CD22-CAR T cell therapies [85]. The patient received an infusion of CAR19-41BB-CD3ζ-mIL15 T cells and was able to achieve a complete response for 5 months, but the tumor did relapse due to a CD19 antigen escape [85]. Despite the heavy tumor burden before mbIL15 CAR T cell infusion, the patient achieved a 5-month complete response with high expansion and long persistence of CAR T cells. The serum level of IL-15 was maintained at a low level with reversible toxicity [85]. This was the first clinical trial to demonstrate that CAR T cells expressing transgenic membrane-bound IL-15 were well tolerated and effective in treating B-ALL (Table 4). Meanwhile, a phase I study reported that administration of CD5-IL15/IL15sushi CAR also led to rapid reduction of malignant cells within 4 weeks post-infusion with grade I CRS and a brief, transient T-cell aplasia in the patient [86] (Table 4). These results suggest that incorporating IL-15 and its receptor complex may be a safe and useful approach to potentiate CAR T cell therapy.

### 5.2. IL-15-Armed NK Cells

Natural Killer (NK) cells are another popular immune cell population for cancer immunotherapy, given their ability to directly lyse tumor cells without antigen priming. Upon activation, NK cells secrete a spectrum of cytokines that differ from those that can cause cytokine release syndrome (CRS), such as interleukin 6 (IL-6). Therefore, NK cells constitute a compelling platform that has favorable therapeutic features and is less likely to mediate severe cytokine-related toxicities, which can overcome many limitations of the current cell therapies. However, one of the obstacles to utilizing NK cells is that they have a short lifespan. Although there were studies supporting the existence of long-lived memory NK cells, the lack of reliable markers to define memory NK cells hindered their selection for immunotherapy. Therefore, ectopically producing IL-15 on NK cells, which benefits NK cell expansion and persistence, has become an attractive strategy for NK cell-based immunotherapy.

Overexpressing IL-15 or IL-15 receptor complex has been evaluated in CD19-CAR [18], CD123-CAR [87,88], and NKG2D-CAR [89] NK cells, with significantly improved NK cell survival rate and enhanced anti-tumor efficacy. The most encouraging results came from the phase I/II clinical trial where cord blood (CB) derived iC9/CAR.19/IL15 CB-NK cells were used for treating refractory B-cell lymphoma or leukemia [18] (ClinicalTrials.gov Identifier: NCT03056339, Table 4). Single infusions at 1e^5^, 1e^6,^ or 10e^6^ per kg of CAR NK cells were tested without developing CRS, neurotoxicity, or graft-versus-host disease (GvHD). Of the 11 patients involved in the study, 73% responded within 30 days after infusion at all dose levels, with the infused CAR NK cells persisting for at least 12 months [18].

However, safety has become a huge concern in IL-15-armored NK cell therapy. In Christodoulou et al., when they tested the engineered CD123 CAR/IL-15 NK cells in two different AML xenograft mouse models, one of the models with the MV-4-11 cell line demonstrated a high level of toxicity that caused lethal conditions [87,88]. This could result from severe inflammation or cytokine release syndrome (CRS) caused by the high level of circulating human pro-inflammatory cytokines accompanied by the dramatic NK cell expansion. In contrast, CAR/IL-15 NK cells in the other model using the MOLM-13 cell line in the same study saw powerful anti-tumor activity without severe toxicity [88].

### 5.3. IL-15 Armored Unconventional T Cells

In addition to employing T and NK cells as the base for cellular therapy, IL-15 has also been explored in combination with unconventional T cells, including gamma delta T (γδT) and invariant natural killer T (iNKT) cells. Unlike conventional T cells that are restricted by major histocompatibility complex (MHC), unconventional T cells recognize non-polymorphic molecules, freeing them from causing GvHD and providing an ideal platform for off-the-shelf cell therapy. Makkouk et al. created a GPC-3-CAR construct with the 4-1BB/CD3 zeta domain and IL-15 to engineer γδT cells [90]. IL-15 secreting CAR-transduced cells showed enhanced proliferation and anti-tumor activity in a HepG2 xenograft mouse model compared to CAR-transduced cells without IL-15 [90]. Similar approaches were taken with iNKT cells to develop treatments for neuroblastoma. Xu et al. developed a disialoganglioside GD2 optimized CAR construct that co-expresses IL-15 [91]. These IL-15 armored GD2-CAR NKT cells moved into clinical trials (ClinicalTrials.gov Identifier: NCT03294954, Table 4) for treating children with relapsed neuroblastoma [91]. The IL-15 armored GD2-CAR NKT cells achieved encouraging results, as the therapeutic cells exhibited enhanced proliferation and homing to tumor sites. The therapy was well tolerated in three patients, showing that it could potentially be a safe and feasible therapy.

## 6. Outlook

Interleukin-15 (IL-15), the pleiotropic cytokine with various biological functions, shows promising impacts on cancer immunotherapy. Although there are encouraging pre-clinical and clinical results indicating that IL-15 can potentially be utilized as a powerful immunotherapeutic agent [92,93], it is important to note that evidence exists showing certain IL-15 isoforms or complexes might also play a pro-tumorigenic role in hematological malignancies [94] or solid tumors [41,95]. Higher levels of sIL-15 or IL-15Rα were detected in the plasma of patients with autoimmune diseases or cancers compared to that in healthy donors, raising the concern that high IL-15 levels may be correlated to poor clinical outcomes [41]. In addition, it is argued that long-term exposure to IL-15 may modulate the tumor microenvironment and promote tumor evasion [41]. Future studies will be necessary to elucidate all the issues. Considering the complexity of IL-15 biology, there is no direct evidence showing IL-15 is the causation of tumor progression or evasion so far. It was demonstrated that soluble IL-15Rα could act as an enhancer of inflammatory cytokines, such as IL-6, TNFα, and IL-17, which can, in turn, lead to tumor evasion [95]. Thus, the combined effects of IL-15 and other cytokines may be the reason for disease progression. It would be very interesting to conduct in vitro and/or in vivo IL-15/IL-15Rα knockout experiments under those pathological circumstances to evaluate the combined effects of multiple cytokines. Future investigation is required to dissect the pleotropic molecular pathways of IL-15 signaling. In addition, the optimal dose for effective anti-tumor responses while limiting toxicity remains to be determined [65]. The IL-15 super agonists like N-803 have an extended half-life compared to recombinant IL-15, but when administered at the high, clinically necessary dosages, they can cause severe adverse events. Currently, several ongoing clinical trials are testing the safety and efficacy of IL-15 derivatives. The results will provide evidence of how to better transform IL-15 into clinical applications.

The combination of IL-15 and adoptive cell transfer has shown remarkable outcomes in treating B-cell lymphoma, AML, neuroblastoma, and glioblastoma. Incorporating IL-15 by itself or together with its IL-15 receptor complex into a CAR design both significantly improved anti-tumor effects on treating various tumors and potentiated T/NK persistence. Most studies did not observe the autonomous growth or leukemic transformation of IL-15 engineered CAR T or CAR NK cells, as only picogram quantities of IL-15 were produced by engineered cells. However, in one of the AML xenograft mouse models, dramatic NK cell expansion correlated with a high level of circulating human pro-inflammatory cytokines potentially led to the lethal death of treated mice, raising concerns about the safety of IL-15 armored immune cells in cell therapy. Meanwhile, regarding the high-grade transient myelotoxicity observed in the CAR19 NK phase I/II trial, it is important to ask whether it was mediated by the lymphodepleting regimen or related to the ectopic expression of IL-15 [18]. While IL-15 exhibits many benefits, these issues need to be further investigated in future studies for IL-15 therapies to move forward.

Another point to consider in NK cell-based therapy is the duration of stimulation with IL-15. Unlike in CD8^+^ T cells that exhibited robust effector functions upon chronic stimulation with IL-15/IL-15Rα complexes, prolonged stimulation to NK cells with IL-15/IL-15Rα complexes could induce impaired activation (i.e., NK hyporesponsiveness) and further alter the balance of activating and inhibitory receptors [96]. Intermittent exposure to IL-15 may be a potential solution to arrest the exhaustion of NK cells [96,97]. In addition, preactivation and restimulation of NK cells with a combination of IL-12, IL-15, and IL-21 cytokines developed a memory-like NK phenotype that could be maintained [98], providing for the potential use of combinatorial cytokines. These facts provide important implications when designing therapeutic strategies for IL-15-based immunotherapies.

In summary, the discovery and progress of IL-15 shifted the field of immunotherapy, revealing advantageous characteristics over other cytokines. While a significant amount of research is still required to address the safety and efficacy of IL-15 in cell-based therapies, we expect to see great advances in utilizing IL-15 in the next few decades.

## Figures and Tables

**Table 1:** IL-15/IL-15Rα isoforms and their specific binding targets.

IL-15 Isoforms	Construct	Binding Targets/Affinity	Cell Specificity	References
sch rhIL-15	*Escherichia coli*-derived IL-15 monomer	IL-15Rα, IL-2/IL-15Rβ/γc	Mature NK cells, NKT cells	[16,23,25]
Receptor-Linker-IL-15 (RLI)	Receptor-Linker-IL-15 fusion protein consisting of IL-15 linked to the sushi domain of IL-15Rα	IL-2/IL-15Rβ/γc	CD8^+^ T cells, NK cells, neutrophils	[16,30,31]
hetIL-15 (NIZ985)	IL-15 heterodimer with soluble IL-15Rα	IL-2/IL-15Rβ/γc	NK cells, CD8^+^ T cells	[32]
hetIL-15FC of human IgG1 (hetIL-15Fc)	A fully glycosylated form of hetIL-15 where the C-terminus of IL-15Rα is covalently linked to the Fc region of human IgG1	IL-2/IL-15Rβ/γc, FcγRs	NK and memory-like CD8^+^ T	[16,33,34,35,36]
N-803 (ALT-803)	An IL-15 super-agonist comprised of an IL-15 variant with N72D mutant complexed with a human IL-15Rα sushi domain-Fc fusion protein	IL-2/IL-15Rβ/γc, FcγRs	NK, CD4^+^ and CD8^+^ memory T-cells	[37,38,39]
NKTR-255	A polyethylene glycol-conjugate of rhIL-15	IL-15Rα, IL-2/IL-15Rβ/γc	Mature NK, CD8^+^ T cells	[40]
Others				
IL-15RαΔ2	Deletion of sushi domain; found on cell membrane despite not binding IL-15	not binding to IL-15		[41]
IL-15RαΔ3	Deletion of linker/hinge region	Moderate affinity to IL-15		[42]
IL-15RαIC3	Directed to secretory pathway to function as a soluble secreted cytokine	High for IL-15		[43]
IL-15Δ6	Induced during immune activation; interferes with IL-15/IL-15Rα super-agonist generation; no effect on T cell proliferation when complexed with IL-15Rα	Lower for IL-15Rα; complex does not bind IL-15Rβ/γc		[44]
IL-15LSP	Directed to secretory pathway by association with golgi apparatus and endoplasmic reticulum	High for IL-15Rα		[8]

Note that there are many other isoforms of these two molecules that exist but aren’t listed because they cause no apparent change in function or they haven’t been well researched enough to determine their function. Δ2, exon 2 deletion; Δ3, exon 3 deletion; IC3, C-terminal insertion between exon 6 and exon 7 that does not contain Ex2A; Δ6, exon 6 deletion; LSP, long signaling peptide (48-aa) consisting of exons 3 through 5.

**Table 2:** Comparison of IL-15 and IL-2 in immunotherapies.

IL-2	IL-15
Stimulation of Tregs due to IL2-Ra presence on Tregs	Lack of stimulation on Treg due to lack of interaction between Treg
Promotes AICD	Suppresses AICD
Increased apoptosis	Reduced apoptosis
Reduced T-cell persistence	Increased T-cell persistence
Higher toxicity profile	Lower toxicity profile
Correlated with CLS	Unassociated with CLS
Displays grade 3 and 4 AEs	Displays grade 1 and grade 2 AEs

Treg, regulatory T cells; AICD, activation-induced cell death; CLS, capillary leak syndrome; AEs, adverse effects.

**Table 3:** Clinical trials of recombinant human IL-15 (rhIL-15) and its derivatives in cancer immunotherapy. i.v., intravenous; s.c., Subcutaneous; ivb, intravenous bolus; CIV, continuous intravenous infusion; BCG, Bacillus Calmette-Geurin. AML, acute myelogenous leukemia; ccRCC, clear-cell renal cell carcinoma; NSCLC, non-small cell lung cancer; SCHNC, squamous cell head and neck cancer; mRCC, metastatic renal cell cancer; CLL, chronic lymphocytic leukemia; ATL, adult T-cell leukemia; PTCL, peripheral T-cell lymphoma; CTCL, cutaneous T-cell lymphoma; ALCL, anaplastic large cell lymphoma; ALL, acute lymphoblastic leukemia; MDS, myelodysplastic syndromes; CML, chronic myelogenous leukemia; MM, multiple myeloma; GEJC, gastroesophageal junction cancers; HNSCC, advanced head and neck squamous cell carcinoma; NMIBC, non-muscle-invasive bladder cancer; mPC, metastatic prostate cancer; SCLC, small cell lung cancer; MCC, merkel cell carcinoma; NHL, non-Hodgkin lymphoma; CRC, colorectal carcinoma; sSCC, cutaneous squamous cell carcinoma; ASCC, anal cell carcinoma.

Clinical Trial	Agent	Description	Malignancies	Institution
NCT01385423	rhIL-15	i.v. IL-15 in combination with haploidentical donor NK cells	AML; Myelodysplastic Syndrome	Masonic Cancer Center, University of Minnesota
NCT02395822	rhIL-15	s.c. IL-15 in combination with donor IL-15 activated NK cells	AML	Masonic Cancer Center, University of Minnesota
NCT04150562	rhIL-15	i.v. IL-15 in combination with avelumab	ccRCC	National Cancer Institute
NCT01727076	rhIL-15	Efficacy and dose escalation study of IL-15 administered s.c.	Advanced Melanoma; Kidney Cancer; NSCLC; SCHNC	National Cancer Institute
NCT01021059	rhIL-15	Safety and dose escalation study of Il-15 (i.v.)	Metastatic Malignant Melanoma; mRCC	National Cancer Institute
NCT01369888	rhIL-15	Safety and dosage study of IL-15 administered (ivb) following lymphodepleting chemotherapy and adoptive cell transfer of TILs	Metastatic Melanoma	National Cancer Institute
NCT01572493	rhIL-15	Continuous intravenous infusion (CIV) of IL-15	Lymphoma; Carcinoma	National Cancer Institute
NCT03759184	rhIL-15	CIV of IL-15 in combination with obinutuzumab	CLL	National Cancer Institute
NCT04185220	rhIL-15	CIV of IL-15 in combination with mogamulizmab	Adult T-cell Lymphoma/Leukemia; Sezary Syndrome; Mycosis Fungoides	National Cancer Institute
NCT03388632	rhIL-15	Safety and dosage study of IL-15 (s.c.) in combination with checkpoint inhibitors nivolumab or ipilimumab or both	Metastatic Solid Tumors; Treatment-Refractory Cancers	National Cancer Institute
NCT02689453	rhIL-15	Dosage, safety, and efficacy study of IL-15 (s.c.) in combination with alemtuzumab	Relapsed T-cell Lymphoma; ATL; PTCL; CTCL; T-cell Prolymphocytic Leukemia	National Cancer Institute
NCT03905135	rhIL-15	CIV of IL-15 in combination with avelumab (Bacenico)	Peripheral T-cell Lymphoma; Mycosis Fungoides; Sezary Syndrome; ALCL	National Cancer Institute
NCT01875601	rhIL-15	Toxicity and dose escalation study of IL-15 in combination with NK cell infusion following lymphodepletion, analysis of pharmacokinetics in pediatric patients, and anti-tumor efficacy	Solid Tumors; Brain Tumors; Sarcoma; Pediatric Cancers; Neuroblastoma	National Cancer Institute
NCT02452268	NIZ985	Dose escalation and expansion study oNIZ985 and NIZ985 (s.c.) in combination with PDR001	Metastatic and Advanced Solid Tumors	Novartis Pharmaceuticals
NCT02452268	NIZ985	Dose escalation and expansion study of NIZ985 and NIZ985 (s.c.) in combination with spartalizumab	In escalation: solid tumors and lymphoma In expansion: melanoma	Novartis Pharmaceuticals
NCT01885897	N-803	Dose escalation and extended study of ALT-803 (i.v.)	AML; ALL; MDS; Lymphoma;CLL; CML	Masonic Cancer Center, University of Minnesota
NCT02099539	N-803	Dose escalation study of N-803 (i.v. vs. s.c.)	Relapsed or Refractory MM	Altor BioScience
NCT03853317	N-803	N-803 in combination with off-the-shelf CD16 targeted NK cells (haNK) and avelumab	Merkel Cell Carcinoma	ImmunityBio Incorporated
NCT02989844	N-803	N-803 for the maintenance after allogeneic hematopoietic cell transplant (alloHCT)	AML; MDS	Masonic Cancer Center, University of Minnesota
NCT04847466	N-803	Efficacy study of irradiated PD-L1 CAR-NK cells combined with pembrolizumab and N-803 (s.c.)	GEJ; HNSCC	National Cancer Institute
NCT03022825 NCT02138734	N-803	BCG in combination with N-803 or BCG alone or N-803 alone administered via intravesical instillation	NMIBC	ImmunityBio Incorporated
NCT04247282	N-803	TriAd vaccine in combination with N-803 (s.c.)	Head and Neck Cancer Head and Neck Neoplasms	National Cancer Institute
NCT03493945	N-803	2-. 3-, or 4 4-drug combinations of M7824, BN-brachyury vaccine, N-803 and Epacodstat	mPC; Prostate Cancer; Prostate Neoplasm; Solid Tumors	National Cancer Institute
NCT03520686	N-803	N-803 (s.c.) in combination with either pembrolizumab, carboplatin + nab-paclitaxel + pembrolizumab, or cisplatin + carboplatin + nab-paclitaxel + pembrolizumab	NSCLC	ImmunityBio Incorporated
NCT05096663	N-803	N-803 (s.c.) in combination with pembrolizumab in comparison to standard care therapy a	Advanced NSCCLC	Southwest Oncology Group
NCT04491955	N-803	N-803 (s.c.) in combination with CV301 vaccine, M7824, and NHS-IL12	Small Bowel Cancers Colorectal Cancers	National Cancer Institute
NCT04927884	N-803	N-803 in combination with sacituzumab	Advanced Triple Negative Breast Cancer	ImmunityBio Incorporated
NCT04898543	N-803	N-803 (s.c.) in combination with memory-cytokine enriched NK (m-ceNK) cells	Metastatic Solid Tumors	ImmunityBio Incorporated
NCT04290546	N-803	N-803 (s.c.) in combination with cytokine-induced memory-like (CIML) NK-enriched cells	HNSCC	Dana-Farber Cancer Institute
NCT03003728	N-803	N-803 (s.c.) in combination with elotuzumab, melphalan, and expanded NK cell autologous stem cell transplantation	MM	University of Arkansas
NCT02559674	N-803	Dose escalation study of N-803 (s.c.) in combination with gemcitabine and nab-paclitaxel	Advanced Pancreatic Cancer	Altor BioScience
NCT03228667	N-803	PD-1/PD-L1 checkpoint inhibitors in combination with N-803 and subsequently combined with PD-L1t-haNK cell therapy in patients with prior treatment of PD-1/PD-L1 checkpoint inhibitors	NSCLC; SCLC; Urothelial Carcinoma; HNSCC; MCC; Melanoma; RCC; Gastric Cancer; Cervical Cancer; and others	ImmunityBio Incorporated
NCT04659629	NL-201	Safety study of NL-201 (i.v.) alone or in combination with pembrolizumab	Solid Tumor Cancers	Neoleukin Therapeutics Incorporated
NCT04136756	NKTR-255	NKTR-255 (i.v.) IL-15 receptor agonist alone or in combination with daratumumab or rituximab	MM; NHL; Indolent Non-Hodgkin Lymphoma	Nektar Theraputics
NCT05327530	NKTR-255	NKTR-255 (i.v.) IL-15 receptor agonist in combination with avelumab	Locally Advanced or Metastatic Urothelial Carcinoma	EMD Serono Research and Development Institute Incorporated
NCT04616196	NKTR-255	NKTR-255 (i.v.) IL-15 receptor agonist alone or in combination with cetuximab	HNSCC; CRC; cSCC; ASCC; Cerivcal Cancer	Nektar Theraputics
NCT05359211	NKTR-255	NKTR-255 (i.v.) IL-15 receptor agonist in combination with autologous CD19-CAR T cells	B-Cell Lymphoma	Fred Hutchinson Cancer Center

**Table 4:** Clinical trials of IL-15 armored cells in cancer immunotherapy. IL-21, Interleukin-21; iC9; iCaspase9; IL-7, Interleukin-7; RMS, rhabdomyosarcoma; HCC, hepatocellular carcinoma; HBL, hepatoblastoma; B-ALL, B-cell acute lymphoblastic leukemia; ALL, acute lymphocytic leukemia; NHL non-Hodgkin’s lymphoma; PMBCL, Primary mediastinal large B-cell lymphoma; DLBCL, diffuse large B-cell lymphoma; FL, follicular lymphoma; MCL, mantle cell lymphoma; SLL, small lymphocytic lymphoma; NB, neuroblastoma; OS, osteosarcoma; CLL, chronic lymphocytic leukemia.

Clinical Trial	Agent	Description	Malignancies	Institution
NCT04377932 NCT05103631	GPC3-CAR IL-15 armored T cells	GPC3-CAR T cells armored with IL-15	Liver Cancer; RMS; Malignant Rhabdoid Tumor; Liposarcoma; Wilms Tumor; Yolk Sac Tumor	Baylor College of Medicine
NCT04715191	GPC3-CAR IL-15 and IL-21 armored T cells	GPC3-CAR T cells armored with IL-15 and IL-21	Liver Cancer; RMS; Malignant Rhabdoid Tumor; Liposarcoma; Wilms Tumor; Yolk Sac Tumor	Baylor College of Medicine
NCT04093648	GPC3-CAR IL-15 and IL-15/IL-21 armored T cells (TEGAR)	Safety and dosage study of GPC3-CAR T cells co-expressing IL-15 or both IL-15 and IL-21	HCC HBL	Baylor College of Medicine
NCT04844086	CD19-mbIL15-CAR T cells	Safety, efficacy, and dosage study of RPM CD19 mbIL-15 CAR T cells	B-ALL; Relapsed/Refractory NHL; PMBCL; DLBCL; FL; MCL; High-grade B-Cell Lymphoma	Eden BioCell Ltd.
NCT05110742	CD5 CAR IL-15 Transduced NK Cells	Dosage and efficacy study for CAR5/IL-15-transduced cord blood-derived NK cells	T-cell malignancies; MCL; CLL	M.D. Anderson Cancer Center
NCT05092451	CAR.70/IL-15 transduced NK cells	Dosage and efficacy study for CAR70/IL-15-transduced cord blood-derived NK cells	B-cell lymphoma; MDS; AML	M.D. Anderson Cancer Center
NCT03774654	CD19.CAR NKT (ANCHOR) cells	Safety, efficacy, and dose escalation study of allogeneic CD19 CAR NKT cells co-expressing IL-15 (ANCHOR)	Refractory B-NHL; Refractory B-Cell SLL; Relapsed Adult ALL; Relapsed CLL; Relapsed NHL	Baylor College of Medicine
NCT02652910	IL-7/IL-15 armored CD19-CAR T cells	Efficacy and persistence study of IL-7/IL-15 armored CD19-CAR T cells	Recurrent Adult DLBCL; Recurrent FL; Recurrent MCL; Stage III & IV Adult DLBCL; Stage III & IV FL; Stage III & IV MCL	Xinqiao Hospital of Chongqing
NCT03721068	iC9.GD2.CAR.IL-15 T cells	Safety and dosage study of GD2-CAR T cells co-expressing IL-15 and iC9	NB; OS	University of North Carolina Lineberger Comprehensive Cancer Center
NCT04814004	hCD19.IL15.CAR iNKT cells	Safety and efficacy study of hCD19.CAR invariant natural killer T cells	ALL; B-cell Lymphoma; CLL	Xuzhou Medical University
NCT03579927	CD19-CD28-zeta-2A-iCasp9-IL15 NK cells	Toxicity and dosage study of CD19-CD28-zeta-2A-iC9-IL-15 NK cells	MCL; Recurrent/refractory DLBCL; Recurrent/refractory FL; Recurrent/refractory B-Cell NHL	M.D. Anderson Cancer Center
NCT03056339	iC9/CAR.19/IL15-Transduced CB NK Cells	Efficacy and dosage study of umbilical and cord blood (CB) derived iC9/CAR.19/IL-15 transduced NK cells	B-Lymphoid Malignancies; ALL; CLL; NHL	M.D. Anderson Cancer Center
NCT05334329	NK cells co-expressing PD-L1 and IL-15	Safety, dosage, and persistence study of COH06 umbilical cord blood-derived NK cells co-expressing PD-L1 and IL-15 alone or in combination with atezolizumab	Advanced/metastatic/refractory NSCLC; Stage III, IIIA, IIIB, IIIC, IV, IVA, and IVB Lung Cancer	City of Hope Medical Center

## Data Availability

Not applicable.
